# Surgical treatment of pulmonary artery sling and tracheal stenosis with or without tracheoplasty: A protocol for a systematic review and meta-analysis

**DOI:** 10.1097/MD.0000000000017449

**Published:** 2019-10-04

**Authors:** Dongxu Li, Xu Zhou, Mengsi Li, Xiaojing Liu

**Affiliations:** a Department of Cardiovascular Surgery; b Evidence-based Medicine Research Center, School of Basic Medical Sciences, Jiangxi University of Traditional Chinese Medicine, Nanchang; c Department of Anesthesiology; d Laboratory of Cardiovascular Disease, West China Hospital, Sichuan University, Chengdu, PR China.

**Keywords:** meta-analysis, protocol, pulmonary artery sling, repair, tracheal stenosis, tracheoplasty

## Abstract

**Background:**

Pulmonary artery sling (PAS) is rare, often with tracheal stenosis. And the postoperative mortality is high. For now, there is no consensus on the tracheoplasty for the patients with PAS and tracheal stenosis.

**Methods:**

Studies involving surgical repair of PAS and tracheal stenosis with and without tracheoplasty were identified by searching the PubMed, EMBASE, and the Cochrane Library databases until June 5, 2019. The assessed variables included ventilation time, early and late mortality, and symptom at follow-up. A random-effect/fixed-effect model was used to summarize the estimates of the mean difference (MD)/risk ratio (RR) with 95% confidence interval (CI).

**Results:**

This study will be submitted to a peer-reviewed journal for publication.

**Conclusion:**

This study will assess the safety and efficacy of tracheoplasty for patients with PAS and tracheal stenosis, and provide more evidence-based guidance in clinical practice.

**PROSPERO registration number:**

CRD42019139788.

## 1 Introduction

Pulmonary artery sling (PAS) is a rare congenital heart disease in which the left pulmonary artery (LPA) originates from the right pulmonary artery and encircles the distal trachea and right main stem bronchus.^[1]^ Patients with PAS frequently have some respiratory symptoms, due to extrinsic tracheal compression by the anomalous LPA with or without intrinsic severe diffuse tracheal stenosis by complete cartilage rings.^[2]^ And the ring-sling cases account for about 65% of patients with PAS.^[3]^ Echocardiography, computed tomography reconstruction, and bronchoscopy are used for the diagnosis and surgical plans.^[4]^

Surgical treatments have evolved toward using cardiopulmonary bypass in most cases and a median sternotomy.^[5]^ The anomalous LPA is generally divided and reimplanted into the main pulmonary artery, and coexisting intracardiac anomalies are repaired simultaneously.^[6]^ With respect to the strategy of dealing with the tracheal stenosis, there are still controversial.^[7]^ The concerns arise from the evidence that tracheoplasty might increase the risk of death or complications. Moreover there are a growing number of reports showing encouraging results with conservative management.^[8]^

Therefore, we aimed to perform this meta-analysis to assess the safety and efficacy of tracheoplasty as part of the surgical treatments for patients with PAS and tracheal stenosis, and hope to support evidence for clinical strategy.

## 2 Methods

### 2.1 Study registration

This protocol is conducted according to the Preferred Reporting Items for Systematic Review and Meta-Analysis Protocols (PRISMA-P) statement.^[9]^ We will report the results of this systematic review and meta-analysis adhere to the Preferred Reporting Items for Systematic Reviews and Meta-Analysis (PRISMA) guidelines.^[10]^ This protocol has been registered in the PROSPERO network (registration number: CRD42019139788).

### 2.2 Ethics and dissemination

#### 2.2.1 Ethics issues

This meta-analysis is a secondary research which based on some previously published data. Therefore, the ethical approval or informed consent was not required in this meta-analysis.

#### 2.2.2 Publication plan

This meta-analysis will be published in a peer-reviewed journal.

### 2.3 Eligibility criteria

#### 2.3.1 Types of studies

Randomized controlled trials (RCTs) and non-RCTs will be incorporated in this study without published year, publication status limitations.

#### 2.3.2 Types of participants

The patients diagnosed with PAS and tracheal stenosis by computed tomography will be included. There will be no restrictions on sex, ethnicity, economic status, and education.

#### 2.3.3 Types of interventions and comparators

The treatment group will be treated with PAS repair and tracheoplasty. According to Backer et al, tracheoplasty has included pericardial patch tracheoplasty, tracheal autograft, tracheal resection, and slide tracheoplasty.^[11]^ The control group will be treated with PAS repair only.

#### 2.3.4 Types of outcome measures

The primary outcomes are ventilation time measured in ICU, early mortality counted in hospital, and late mortality counted out of hospital. The secondary outcome is respiratory symptom at follow-up.

### 2.4 Search strategy

We will search PubMed (Medline), EMBASE, and the Cochrane Library databases for related studies published without language restrictions before June 5, 2019. We will use the relevant keywords or subject terms adhered to Medical Subject Heading terms to search for eligible studies in the electronic databases which were mentioned above without language restrictions. The PubMed search strategies are “pulmonary artery sling[tw] and (surgery[tw] or operation[tw] or procedure[tw] or treatment[tw] or management[tw] or tracheoplasty[tw] or tracheal[tw]or repair[tw])”.

### 2.5 Data collection and analysis

#### 2.5.1 Data management

Endnote X7 software (Thomson Reuters, Canada) will be used for literature managing and records searching. A pilot-test will be conducted to ensure the inter-rater is reliability between the reviewers before the literature selection.

#### 2.5.2 Study selection

Two reviewers (DXL, XZ) will investigate each title and abstract of all literatures searched independently and identify whether the trials meet the inclusion criteria as designed and described in this protocol. Two authors (DXL, XZ) will in duplicate and independently screen the full text of all potential eligible studies to exclude irrelevant studies or determine eligibility. The 2 reviewers will list all the studies included and document the primary reasons of exclusion for studies that do not conform to the inclusion criteria. Disagreements between the 2 authors will be resolved by discussing with the third author (MSL), if necessary, consulting with the fourth author (XJL). We will show the selection process in details in the PRISMA flow chart.^[9]^

#### 2.5.3 Data collection process

Two independent researchers extracted data (DXL, XZ). Any disagreements will be resolved by a third reviewer (XZ). The following data were extracted from each eligible study using a standardized data collection form: first author's name, study design, publication year, country where the study was conducted, sample size, age, weight, gender, main diagnosis, cardiac anomaly, tracheal stenosis of total length, intubation before surgery, complete cartilage rings, type of tracheoplasty, and follow-up interval. Data were collected for ventilation time after surgery, early and late death, and symptom at last follow-up.

### 2.6 Quality of evidence assessment

According to Grading of Recommendations Assessment Development and Evaluation (GRADE), the quality of included studies will be assessed by the online guideline development tool http://gdt.guidelinedevelopment.org/), and divided into 4 levels: high quality, moderate quality, low quality, and very low quality.^[12]^

### 2.7 Assessment of risk of bias in included studies

The methodological quality of the included studies was assessed by 2 authors (DXL, MSL). The Cochrane Risk of Bias tool, which contains 7 specific domains: random sequence generation (selection bias); allocation concealment (selection bias); blinding of participants and personnel (performance bias); blinding of outcome assessment (detection bias); incomplete outcome data (attrition bias); selective outcome reporting (reporting bias); other bias, will be used to assess the methodological quality of RCTs as low risk, high risk, or unclear risk of bias.^[13]^ If any domain is scored high/low risk of bias, the study will be considered high/low risk of bias.

Nine-item Newcastle–Ottawa Quality scale, is a widely used tool to assess quality of non-randomized trials by risk evaluation of adequacy of selection, comparability, and outcomes assessment.^[14]^ The high-quality study was defined as a study with ≥ 6 scores.

### 2.8 Data analysis

The measures of the effects of interest were the risk ratio (RR)/mean difference (MD) with 95% confidence intervals (CI). A subgroup analysis was performed stratified by patients with tracheal rings. We used the Cochrane Chi-Squared test (*Q* test) and the *I*^2^ test to evaluate level of heterogeneity across studies. When significant heterogeneity (*P* < .05 or *I*^2^ > 50%) was detected, we pooled data using a random-effect model.^[15]^ Otherwise, a fixed-effect model was used. We explored the source of heterogeneity using sensitivity analysis. Funnel plots were visually inspected to identify any potential publication bias. All statistical analyses were performed using Stata software (version 14.0; Stata Corp., College Station, TX, USA).

#### 2.8.1 Subgroup analysis

If there is high heterogeneity and the data are sufficient, subgroup analysis will be conducted to search potential causes of heterogeneity. Subgroup analysis will be performed in type of tracheal stenosis.

#### 2.8.2 Sensitivity analysis

We will conduct sensitivity analyses of the primary results to explore the robustness of the review conclusions if feasible, after in consideration of impact of methodological quality, missing data, and sample size.

#### 2.8.3 Publication bias

According to Cochrane Handbook, when enough original studies are included (generally > 10 trials), publication bias analysis will be performed through funnel plot.^[16]^ Symmetrical funnel plot indicates low publication bias, otherwise high risk.

## Author contributions

**Data curation:** Dongxu Li, Xu Zhou.

**Formal analysis:** Dongxu Li, Xu Zhou, Mengsi Li.

**Methodology:** Dongxu Li, Xu Zhou.

**Project administration:** Xiaojing Liu.

**Supervision:** Xiaojing Liu.

**Writing – original draft:** Dongxu Li.

**Writing – review & editing:** Dongxu Li, Mengsi Li, Xiaojing Liu.
